# Intracellular repair of oxidation-damaged α-synuclein fails to target C-terminal modification sites

**DOI:** 10.1038/ncomms10251

**Published:** 2016-01-25

**Authors:** Andres Binolfi, Antonio Limatola, Silvia Verzini, Jonas Kosten, Francois-Xavier Theillet, Honor May Rose, Beata Bekei, Marchel Stuiver, Marleen van Rossum, Philipp Selenko

**Affiliations:** 1 Department of NMR-supported Structural Biology, In-Cell NMR Laboratory, Leibniz Institute of Molecular Pharmacology (FMP Berlin), Robert-Roessle Strasse 10, Berlin 13125, Germany; 2 Department of Pharmacy, University of Naples 'Federico II', Via Domenico Montesanto 49, Naples 80131, Italy.

## Abstract

Cellular oxidative stress serves as a common denominator in many neurodegenerative disorders, including Parkinson's disease. Here we use in-cell NMR spectroscopy to study the fate of the oxidation-damaged Parkinson's disease protein alpha-synuclein (α-Syn) in non-neuronal and neuronal mammalian cells. Specifically, we deliver methionine-oxidized, isotope-enriched α-Syn into cultured cells and follow intracellular protein repair by endogenous enzymes at atomic resolution. We show that N-terminal α-Syn methionines Met1 and Met5 are processed in a stepwise manner, with Met5 being exclusively repaired before Met1. By contrast, C-terminal methionines Met116 and Met127 remain oxidized and are not targeted by cellular enzymes. In turn, persisting oxidative damage in the C-terminus of α-Syn diminishes phosphorylation of Tyr125 by Fyn kinase, which ablates the necessary priming event for Ser129 modification by CK1. These results establish that oxidative stress can lead to the accumulation of chemically and functionally altered α-Syn in cells.

## Abstract (web summary)

α-synuclein is a protein linked to the occurrence of Parkinson's disease. Here, the authors use time-resolved in-cell NMR spectroscopy to study the repair of methionine-oxidized α-synuclein by endogenous cellular enzymes.

## Graphical abstract

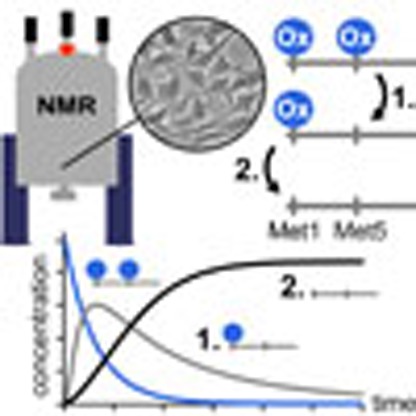

Aggregation of the intrinsically disordered protein alpha-synuclein (α-Syn) into amyloid-rich Lewy bodies is a central event in Parkinson's disease (PD)^1^,^2^. Although PD has a multifactorial aetiology, ageing, reactive oxygen species (ROS) imbalance and cellular oxidative stress constitute common disease hallmarks^3^,^4^. Observations that α-Syn oligomerization directly impairs mitochondrial function and results in the accumulation of ROS suggest that aggregation and cellular oxidative stress are functionally connected^5^. In addition, oxidative α-Syn modifications promote its aggregation *in vitro* and *in vivo*^6^,^7^. Thus, a negative cascade may operate under oxidative stress conditions^8^. In further support of such a model, Lewy body deposits of α-Syn contain an abundance of oxidative modifications, such as nitrated tyrosines and oxidized methionines^9^,^10^,^11^,^12^. Indeed, exposed methionine side-chains are highly oxidation-prone and readily react with a number of physiological oxidants, such as hydroxyl radicals (OH^.^), superoxide anions (O_2_^−^), hydrogen peroxide (H_2_O_2_), chloramines and peroxynitrites^13^. α-Syn contains four methionines, two of which are at its N-terminus (that is, Met1 and Met5) and two are within the C-terminal portion of the protein (that is, Met116 and Met127). Oxidation of methionine residues produces methionine sulfoxides, which display two diastereoisomers, MetO(R) and MetO(S) (ref. 14). The establishment of methionine sulfones, with two oxygens per side-chain sulfur, requires harsh oxidative conditions and is not commonly observed^15^. In the presence of H_2_O_2_, all four α-Syn methionines are readily converted into sulfoxides^16^. In turn, methionine oxidation triggers the formation of intermediate oligomer species that display different degrees of cytotoxicity^17^,^18^,^19^. In PC12 cells, and in cultured primary dopaminergic neurons, exposure to the electron transport chain inhibitor Rotenone^20^ results in α-Syn methionine oxidation and the formation of intracellular inclusions^6^,^21^. Other α-Syn interactions also produce distinct methionine oxidation patterns. Binding to copper or oxidized lipids converts Met1 and Met5 to sulfoxides^22^,^23^, whereas interactions with the neurotransmitter dopamine oxidizes Met127 (ref. 19). α-Syn methionine oxidation decreases its affinity for biological membranes^23^ and impairs degradation by the 20S proteasome, both *in vitro* and in cells^24^.

To counteract the accumulation of oxidation-damaged proteins, cells contain sophisticated repair machineries, such as the family of methionine sulfoxide reductase (MSR) enzymes^25^. In humans, two classes of MSR enzymes exist: MSRA selectively reduces MetO(S) diastereoisomers, whereas MSRB converts MetO(R)^26^,^27^. In addition, different organelle-specific MSR isoforms are present^26^,^27^. Loss of MSR activity results in augmented brain pathologies associated with neurodegenerative disorders, such as Alzheimer's disease and PD^28^,^29^,^30^, and MSRs are thought to exert general protective effects against α-Syn aggregation and cellular oxidative stress-induced apoptosis^31^.

Although methionine-oxidized α-Syn is a known MSRA substrate^31^, the mechanism by which MSRA repairs oxidation-damaged α-Syn is unknown. Therefore, we set out to investigate the fate of methionine-oxidized α-Syn in intact mammalian cells at atomic resolution using time-resolved in-cell nuclear magnetic resonance (in-cell NMR) spectroscopy. We find that endogenous cellular enzymes efficiently process modified Met1 and Met5 of α-Syn, whereas Met116 and Met127 remain oxidized. N-terminal α-Syn repair proceeds in a strictly stepwise manner, with Met5 being processed before Met1 in all tested cell lines. The inability to reduce C-terminal methionine sulfoxides results in the accumulation of irreversibly altered α-Syn species that exhibit impaired phosphorylation of Tyr125 by the major tyrosine kinase Fyn. These results suggest that oxidative damage at Met116 and Met127 modulates the post-translational phosphorylation behaviour of α-Syn in cells.

## Results

### Methionine-oxidized α-Syn exhibits reduced residual helicity

**Figure 1:**
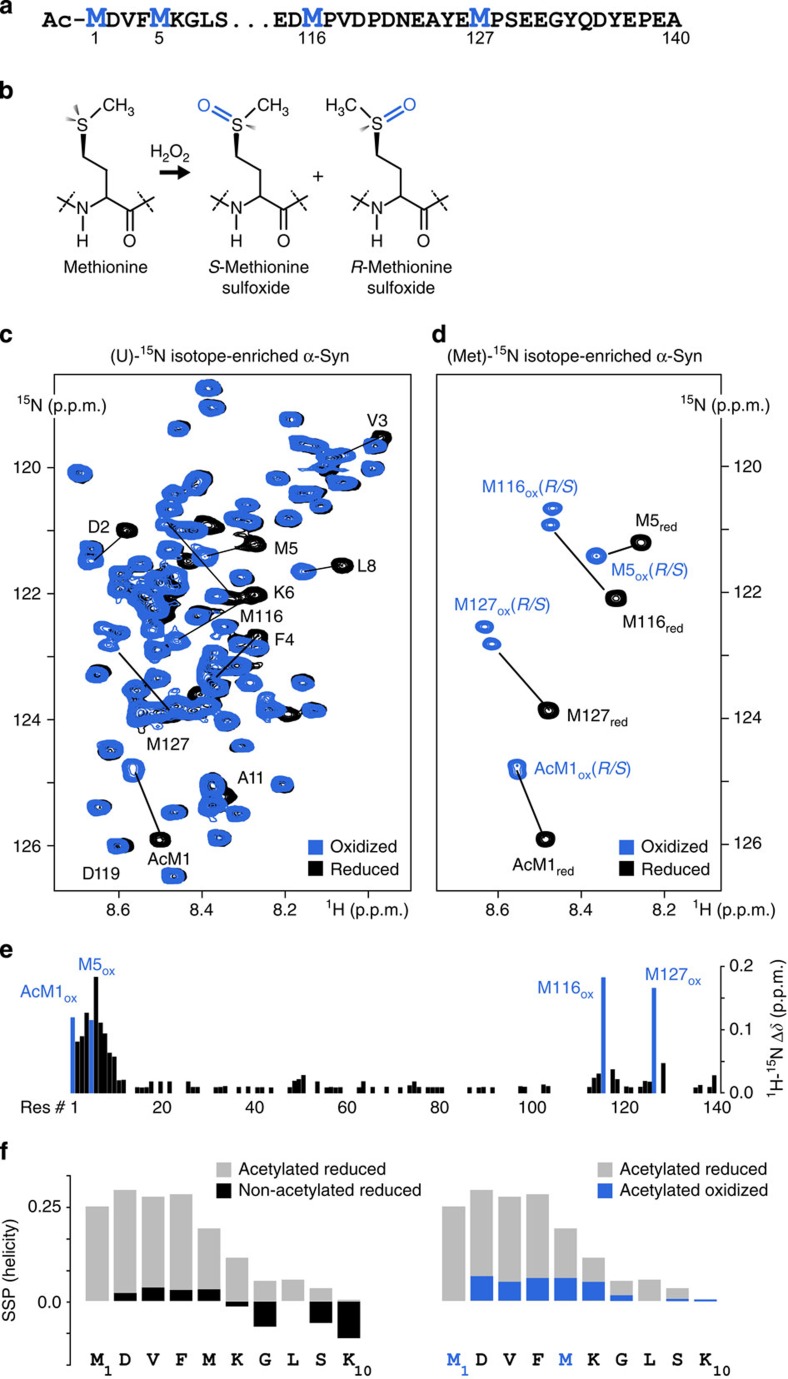
NMR characteristics of methionine-oxidized α-Syn. (**a**) α-Syn amino-acid sequence and highlighted positions of N- and C-terminal methionines. (**b**) Chemistry and stereochemistry of methionine oxidation and sulfoxide formation. (**c**) Overlay of 2D NMR spectra of reduced (black) and oxidized (blue) uniform (U)-^15^N isotope-enriched, N-terminally acetylated α-Syn and of (**d**) methionine-selective (Met-^15^N) isotope-enriched, N-terminally acetylated α-Syn. (**e**) Residue-resolved ^1^H–^15^N mean-weighted chemical shift difference (Δ*δ*) map of reduced versus oxidized α-Syn. Residues Met1, Met5, Met116 and Met127 are shown in blue. (**f**) The SSP scores of the first 10 α-Syn residues in their reduced non- (black) and N-terminally acetylated (grey) forms are shown on the left. SSP scores of acetylated reduced (grey) and acetylated oxidized (blue) α-Syn are shown on the right. SSP scores are calculated based on experimentally determined ^13^Cα and ^13^Cβ chemical shift values.

To determine the fate of oxidation-damaged α-Syn in mammalian cells, we initially reacted uniform (U)-^15^N isotope-enriched, N-terminally acetylated protein^32^ with 4% H_2_O_2_ as outlined previously^16^. This procedure converts all four α-Syn methionines into sulfoxides (Fig. 1a,b). We used NMR spectroscopy to verify that complete oxidation of Met1, Met5, Met116 and Met127 did not alter the overall monomeric, disordered conformation of isolated α-Syn *in vitro* (Fig. 1c), which we independently confirmed using size exclusion chromatography (SEC), circular dichroism (CD) spectroscopy and dynamic light scattering (DLS; Supplementary Fig. 1a–d). To better resolve individual methionine NMR signals, we also produced methionine-selective ^15^N isotope-enriched α-Syn. Two-dimensional (2D) NMR spectra of oxidized Met-^15^N α-Syn revealed two well-resolved amide resonance cross-peaks for Met116 and Met127, as expected for a racemic mixture of R and S diastereoisomers (Fig. 1d). We detected greater R/S chemical shift dispersions for Met116 and Met127 than for Met1 and Met5, which possibly indicates different local conformations of C- versus N-terminal oxidized α-Syn methionines^33^, or reflects sequence-specific effects. Indeed, Met116 and Met127 are both followed by proline residues, whereas Met1 and Met5 are not. Chemical shift difference (Δ*δ*) mapping between reduced and methionine-oxidized α-Syn revealed more pronounced effects in the N-terminus of the protein (Fig. 1e), which pointed towards greater structural alterations in response to oxidation of Met1 and Met5 than to that of Met116 and Met127. By measuring Cα and Cβ chemical shifts and calculating secondary structure propensity (SSP) scores, we found that methionine oxidation greatly diminished the increase in residual α-Syn helicity that occurs in response to physiological N-terminal acetylation^34^,^35^ (Fig. 1f). This may, in turn, explain the reduced membrane-binding affinity of oxidized α-Syn observed by us and others^23^ (Supplementary Fig. 1e,f).

### Oxidized α-Syn methionines 1 and 5 are selectively repaired

**Figure 2:**
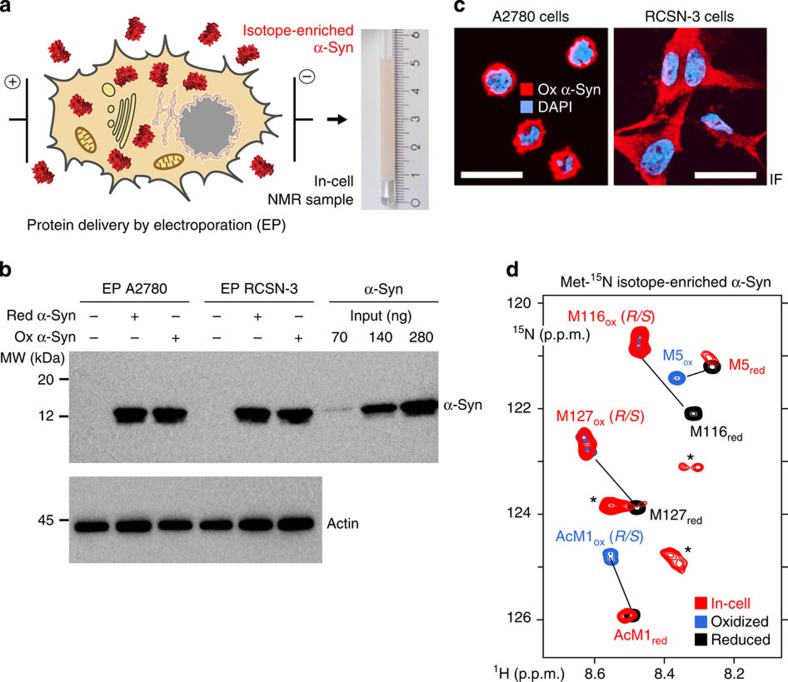
In-cell NMR of methionine-oxidized α-Syn. (**a**) Schematic overview of the electroporation procedure to deliver isotope-labelled α-Syn into mammalian cells and the resulting in-cell NMR sample. (**b**) Western blots of lysates of A2780 and RCSN-3 cells electroporated with reduced or oxidized α-Syn. Reduced, N-terminally acetylated α-Syn was used as the input control. (**c**) Immunofluorescence detection of delivered, oxidized α-Syn in A2780 and RCSN-3 cells after recovery. Scale bars, 50 μm. (**d**) Overlay of 2D in-cell NMR spectra of Met-^15^N isotope-enriched α-Syn in A2780 cells (red), with reference spectra of the reduced (black) and oxidized protein (blue). Asterisks denote metabolite background signals in A2780 cells (see also Supplementary Fig. 4b).

Next, we electroporated oxidized, (U)-^15^N isotope-enriched α-Syn into mammalian A2780 and RCSN-3 cells for high-resolution in-cell NMR measurements. RCSN-3 cells are directly derived from rat *substantia nigra* neurons^36^, the cell type in which α-Syn aggregates are primarily found in PD patients^1^ (Fig. 2a). We assessed successful delivery of α-Syn using western blotting and immunofluorescence imaging, which revealed a uniform cytoplasmic staining of the delivered protein, with no signs of aggregation such as the appearance of bright intracellular foci (Fig. 2b,c and Supplementary Fig. 2). In-cell NMR spectra of oxidized (U)-^15^N α-Syn displayed strong similarities with the disordered reference state of the N-terminally acetylated, monomeric protein (Supplementary Fig. 3a), which established that oxidation-damaged α-Syn remained highly dynamic in A2780 and RCSN-3 cells, and did not stably interact with large cellular structures such as membranes, similar to the reduced form of the protein^37^. We did not detect protein leakage under our experimental conditions (Supplementary Fig. 4a). To better resolve the intracellular oxidation states of α-Syn, we also delivered oxidized methionine-selective ^15^N isotope-enriched α-Syn (Met-^15^N) into A2780 cells and acquired in-cell NMR spectra on the resulting cell samples. We clearly detected the cross-peak pattern of reduced Ac-Met1 and Met5, whereas NMR signals of Met116 and Met127 matched those of the oxidized states of both residues (Fig. 2d). These findings suggested that endogenous activities in A2780 cells processed modified methionines at the N-terminus of α-Syn, but not its C-terminus. To corroborate this hypothesis, we investigated the cross-peak positions of Ac-Met1, Asp2, Met5, Lys6, Leu8 and Ser9 in the in-cell NMR spectra of (U)-^15^N enriched α-Syn, which collectively serve as excellent indicators of the oxidation states of Met1 and Met5 (ref. 23) (Fig. 1c and Supplementary Fig. 1d). Indeed, we found these resonance cross-peaks at positions that matched those of reduced α-Syn (Supplementary Fig. 3a). By contrast, in-cell NMR signals of Met116 and Met127, and of neighbouring residues, confirmed the presence of methionine sulfoxides in the C-terminus of the protein. Cell lysis and direct *in situ* NMR measurements on the resulting slurries further substantiated these findings (Supplementary Fig. 3b). We detected minor degrees of Met127 sulfoxide repair in RCSN-3 cell lysates, as manifested by the presence of two Ser129 cross-peaks corresponding to the oxidized and reduced form of Met127. Together, these results demonstrated that oxidized Met1 and Met5 underwent efficient repair in A2780 and RCSN-3 cells, whereas Met116 and Met127 did not.

### Stepwise repair of N-terminal α-Syn methionine sulfoxides

**Figure 3:**
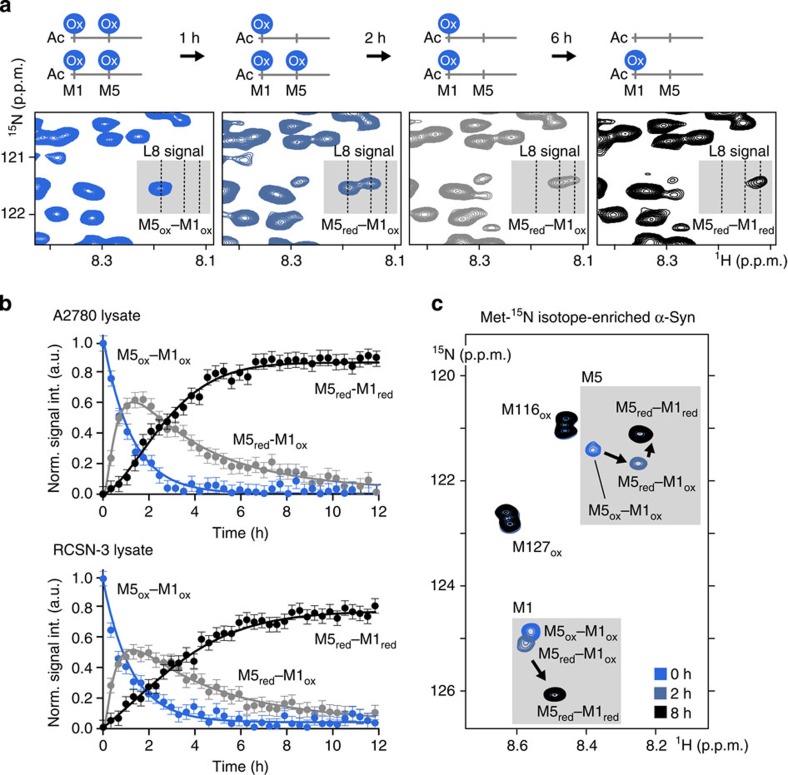
Methionine sulfoxide repair in A2780 and RCSN-3 cell lysates. (**a**) Evolution of A2780 lysate NMR spectra at 0, 1, 2 and 6 h time points having directly added (U)-^15^N isotope-enriched methionine-oxidized, N-terminally acetylated α-Syn and schematic depiction of individual protein species accumulating during the stepwise repair process. Grey boxes highlight characteristic Leu8 chemical shift changes in response to the consecutive reduction of Met5 and Met1. (**b**) Real-time NMR profiles of site-selective sulfoxide repair kinetics in A2780 and RCSN-3 cell lysates. Error bars represent the average experimental noise of the respective 2D NMR spectra. Lysate repair reaction properties were confirmed with two independent samples for each cell line. (**c**) Overlay of time-resolved 2D NMR spectra of Met-^15^N isotope-enriched oxidized, N-terminally acetylated α-Syn in A2780 cell lysate. Grey boxes highlight Met1 and Met5 chemical shift changes during the repair process.

To obtain atomic resolution insights into the selectivity of this repair process, we directly added (U)-^15^N isotope-enriched oxidized α-Syn to lysates of non-electroporated A2780 and RCSN-3 cells and used time-resolved NMR spectroscopy to follow the fate of its oxidized methionines. By delineating the time-dependent changes of NMR signal intensities of the differently modified protein residues, we found that sulfoxide reduction occurred in a stepwise manner with Met5 preceding Met1 in both lysates (Fig. 3a). Time-resolved repair profiles further showed that Met1 was not targeted for as long as oxidized Met5 was present (Fig. 3b), in support of a kinetic model in which oxidized Met5 constitutes the preferred substrate site over modified Met1 (Supplementary Table 1). Lysate NMR experiments with Met-^15^N-oxidized α-Syn confirmed that Met116- and Met127 sulfoxides stably persisted in these physiological environments (Fig. 3c). They further revealed that R and S diastereoisomers of N-terminal methionine sulfoxides were repaired equally well, as inferred from the complete disappearance of oxidized Met1 and Met5 cross-peaks.

Next, we asked whether these properties were similarly displayed in other cell types. To this end, we extended our investigations to HeLa, SK-N-SH, B65 and SH-SY5Y cell lysates. We found that the stepwise repair of Met5 and Met1 occurred in an identical manner in these lysates and with comparable rates, whereas Met116 and Met127 remained oxidized (Supplementary Table 1 and Supplementary Fig. 5).

*In vivo*, methionine sulfoxide reduction depends on the interplay of a multicomponent system that includes enzymes of the methionine sulfoxide reductase family, MSRA and MSRB, and the NADPH-dependent thioredoxin (TRX)/TRX reductase (TRXR) complex (Supplementary Fig. 6a,b)^10^,^11^. To address whether endogenous MSR proteins participated in the observed reactions, we selectively inhibited these enzymes by the addition of increasing amounts of dimethyl sulfoxide^38^,^39^. Time-resolved NMR experiments in A2780 cell lysates revealed progressively diminished repair efficiencies (Supplementary Fig. 6c), which strengthened the notion that cellular MSRs contribute to the processing of N-terminal methionine sulfoxides of oxidation-damaged α-Syn.

### C-terminal methionine sulfoxides impair phosphorylation

**Figure 4:**
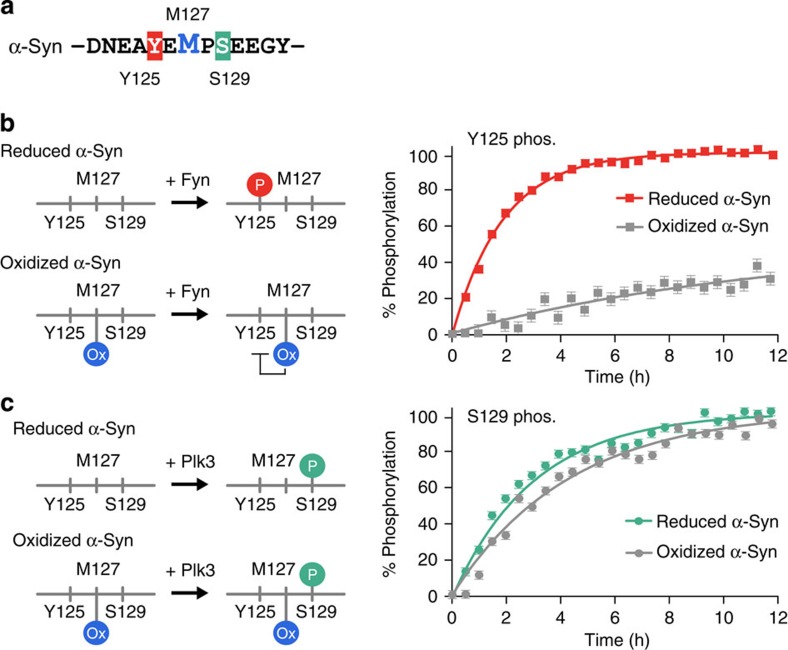
Methionine oxidation and phosphorylation crosstalk. (**a**) The amino-acid sequence of α-Syn residues flanking Met127. Ser129 and Tyr125 phosphorylation sites are highlighted. (**b**) Real-time NMR profiles of Tyr125 phosphorylation kinetics of reduced and methionine-oxidized, N-terminally acetylated α-Syn in reconstituted kinase reactions with recombinant Fyn. (**c**) Real-time NMR profiles of Ser129 phosphorylation kinetics of reduced and methionine-oxidized, N-terminally acetylated α-Syn in reconstituted kinase reactions with recombinant Plk3. Error bars represent the average experimental noise of the respective 2D NMR spectra. Phosphorylation behaviours were confirmed on two independent samples for each kinase.

Given that MSR enzymes failed to repair oxidized methionines at the C-terminus of α-Syn, we asked whether the persistence of these modifications affected the post-translational modification behaviour of α-Syn. We were particularly intrigued by the equidistance of Met127 to Tyr125 and Ser129 (Fig. 4a), two known post-translational phosphorylation sites with implications in PD^40^,^41^. Having shown that phosphorylation of Tyr125 constitutes a necessary priming event for the efficient modification of Ser129 by protein kinase CK1 (ref. 42), we tested whether Met127 oxidation affected the modification of Tyr125. To this end, we reconstituted kinase reactions with recombinant Fyn, the enzyme primarily responsible for Tyr125 phosphorylation *in vivo*^43^,^44^. By recording time-resolved NMR experiments and monitoring of the progressive phosphorylation of different α-Syn sites, we determined that Tyr125 modification of oxidized α-Syn was severely impaired, whereas phosphorylation of Tyr133 and Tyr136 was not affected (Fig. 4b and Supplementary Fig. 7a,b). By contrast, the presence of oxidized C-terminal methionines did not compromise phosphorylation of neighbouring Ser129 by Plk3 (Fig. 4c and Supplementary Fig. 7a), which suggested that the observed effect is kinase-specific and not a general inhibitory feature. These results established that the persistence of methionine sulfoxides in the C-terminus of α-Syn selectively alters the protein's phosphorylation behaviour.

## Discussion

Cellular oxidative stress is causally linked to PD, and high concentrations of ROS are found in the brains of PD patients^3^. Accordingly, intracellular α-Syn aggregates often carry different types of oxidative modifications, such as nitrated tyrosines^45^, di-tyrosines^46^ and oxidized methionines^9^. Several *in vitro* studies further strengthened the notion that methionine oxidation promotes the formation of cytotoxic α-Syn oligomers^17^,^18^,^19^. How these oxidative modifications eventually accumulate in α-Syn amyloid fibrils and Lewy bodies remains unknown.

Methionine oxidation is among the most difficult-to-study post-translational protein modification because sulfoxides are not easily detected with chemical probes or antibodies^29^,^47^ and because they spontaneously form during sample preparation for mass spectrometry^48^,^49^. By contrast, oxidized methionines display unique NMR chemical shift changes that are readily identified in 2D ^1^H–^15^N and ^1^H–^13^C correlation spectra^23^,^50^. For these reasons, NMR spectroscopy has emerged as a powerful tool to study protein methionine oxidation *in vitro*^23^. Here we applied this approach to live cells to delineate the details of intracellular methionine sulfoxide repair at atomic resolution. We demonstrate that endogenous enzymes in A2780 and RCSN-3 cells process oxidized N-terminal α-Syn methionines Met1 and Met5, whereas they do not target modified Met116 and Met127. By showing that these properties are similarly displayed in lysates of HeLa, SK-N-SH, B65 and SH-SY5Y cells, we speculate that they might represent a general mechanism of site-selective α-Syn repair in mammalian cells. In all of our samples, the reduction of oxidized Met5 preceded that of Met1, which supports the notion that sequence-encoded affinity determinants steer the positional selectivity of intracellular repair activities, similar to other enzyme–substrate systems. We did not detect residual NMR signals of methionine-oxidized Met5 and Met1 upon cell lysate incubation and completion of the respective repair reactions, which suggested that R and S diastereoisomers of N-terminal α-Syn sulfoxides are processed with comparable efficiencies. Hence, we concluded that both classes of MSR enzymes participate in these reactions. The presence of MSRA and MRSB proteins in the tested cell lysates strengthens this idea.

At this point, we can only speculate about why these enzymes do not repair C-terminal α-Syn methionine sulfoxides. One explanation might be afforded by the structural context of the entire α-Syn C-terminus, which is thought to exhibit higher degrees of local structure than the N-terminal portion of the protein^33^ and probably restricts access for repair enzymes. A similar explanation has been forwarded as the possible reason for the faster oxidation behaviour of N-terminal α-Syn methionines compared with C-terminal ones^51^,^52^. In agreement with this hypothesis, the measured rates of methionine sulfoxide repair by MSR enzymes scale linearly with the accessibility of individual damage sites^53^. Alternatively, unfavourable electrostatic interactions between negatively charged sequence elements encompassing Met116 and Met127 and enzyme-active sites might cause the observed effects^54^,^55^. Met116- and Met127-adjacent prolines, and their unique backbone geometries, may offer yet another explanation.

Given that C-terminal α-Syn sulfoxides are stably preserved in cells, they likely contribute to the accumulation of permanently altered protein species with possible roles in neurotoxicity^17^,^18^,^19^. Such effects may be mediated by aberrant interactions with proteins that selectively bind to the C-terminus of α-Syn, such as synaptobrevin^56^ and Rab8 (ref. 57), or result in impaired proteasomal degradation and the concomitant accumulation of stable C-terminal fragments that act as dominant-negative modulators of α-Syn's physiological functions^24^. Furthermore, as we have shown here, C-terminal methionine sulfoxides impair phosphorylation of Tyr125 by the major tyrosine kinase Fyn. Indeed, an age- and disease-dependent decline of Tyr125 phosphorylation has been reported for a *Drosophila* model of PD and in humans, although possible correlations with oxidative modifications were not investigated^58^. Because phosphorylated Tyr125 functions as the priming site for the efficient modification of Ser129 by CK1 (ref. 42), reductions in Tyr125 phosphorylation are likely to also diminish CK1-mediated modifications of Ser129, which is supported by recent observations of reduced Ser129 phosphorylation and increased methionine oxidation levels in a *msra*^−/−^ (null) mouse model^59^. However, so far, we only considered the effects of methionine oxidation on kinase activities without taking into account the roles of cellular phosphatases in determining the physiological phosphorylation state(s) of α-Syn^60^. Clearly, the presence of methionine sulfoxides may also impair α-Syn's de-phosphorylation behaviour and its avidity as a phosphatase substrate. Investigations addressing the roles of phosphatases in this context are currently underway.

## Methods

### Recombinant protein expression

^15^N or ^15^N/^13^C isotopically enriched, N-terminally acetylated α-Syn was obtained by co-expressing the wild-type α-Syn plasmid (pT7-7) with the yeast NatB acetylase complex^61^ in *Escherichia coli* BL21 (DE3) Express cells. Bacteria were grown in M9 minimal media supplemented with 0.5 g l^−1^of ^15^N NH_4_Cl (Sigma) and/or 2 g l^−1^ of ^13^C D-glucose (Sigma) until an OD_600_ of 0.6 was reached. After induction with 1 mM isopropyl-β-D-thiogalactoside (Roth), cells were grown for 12 h at 30 °C. For ^15^N/^13^C methionine-selective isotope-enriched samples, M9 medium contained natural abundance of NH_4_Cl, D-glucose and 0.3 mM of ^15^N/^13^C-enriched L-methionine (Isotec, 608106)^62^. α-Syn was purified using a standard purification protocol that includes a 20-min boiling step of the bacterial lysate and selective protein precipitation with ammonium sulfate (360 mg ml^−1^)^34^,^63^. α-Syn was further purified using anion exchange and SEC on an AKTA FPLC machine and Amersham MonoQ and Superdex 75 columns, respectively. Purity was confirmed with SDS–PAGE. Quantitative N-terminal acetylation was assessed using 2D ^1^H–^15^N NMR spectroscopy. Methionine-oxidized, N-terminally acetylated α-Syn was prepared by incubating 500 μM of recombinant protein with 4% H_2_O_2_ (Sigma)^16^ for 2 h and subsequent purification using SEC to remove excess H_2_O_2_. Complete peroxide removal was additionally verified with a colorimetric assay (Enzo Life Sciences). Quantitative methionine oxidation was confirmed using NMR spectroscopy (2D ^1^H–^15^N correlation spectra) in 20 mM phosphate buffer, pH 7.0, 150 mM NaCl and 10% D_2_O (that is, NMR buffer). Concentrations were assessed spectrophotometrically by absorption measurements at 274 nm (*ɛ*=5,600 M^−1 ^cm^−1^).

### CD spectroscopy

CD measurements were performed on a Jasco J-720 spectropolarimeter at 25 °C with 10 μM methionine-oxidized, N-terminally acetylated α-Syn in NMR buffer at pH 7.0. Far-ultraviolet CD spectra were collected using a 0.1-cm path-length cuvette. Eight scans were averaged and blank (buffer) spectra were subtracted from protein spectra to calculate ellipticity (mdeg).

### SEC

Overall, 200 μl of 500 μM methionine-oxidized, N-terminally acetylated α-Syn was separated on a Superdex 75 analytical column (GE Healthcare) connected to an AKTA purifier FPLC machine (GE Healthcare). Absorbance at 280 nm was plotted against elution volumes for SEC profiles.

### DLS

DLS was performed on a Malvern Zetasizer Nano operating at a laser wavelength of 633 nm at 25 °C in 3 × 3 mm cuvettes and with 50 μM of methionine-oxidized, N-terminally acetylated α-Syn in NMR buffer. Hydrodynamic radii were calculated based on volume-weighted analyses of scattered light, using the Malvern DTS software.

### Preparation of small unilamellar vesicles

Small unilamellar vesicles (SUVs) were reconstituted with pig brain polar lipids (Avanti) using freeze–thaw cycles and sonication. Briefly, the lipid powder was resuspended in NMR buffer and vortexed for 30 min at room temperature. Solutions were frozen on dry ice (5 min) and thawed at 37 °C for 5 min, five times before sonication at 4 °C for 20 min at 30% power settings (Bandelin Sonoplus), under a nitrogen stream^64^. Initial stock solutions were adjusted to 20 mM considering the total lipid input. Average vesicle sizes of 60 nm were determined with DLS.

### NMR spectroscopy

For NMR resonance assignment of reduced and fully methionine-oxidized α-Syn, we used ^15^N/^13^C isotope-enriched (500 μM) protein in NMR buffer at pH 7.0. 2D (^1^H–^15^N) SOFAST-HMQC (ref. 65) and three-dimensional (3D) triple resonance HNCACB and CBCACONH experiments (Bruker standard pulse sequences) were recorded at 15 °C on a 750-MHz Bruker Avance spectrometer, equipped with a cryogenically cooled proton optimized triple resonance >‘inverse' NMR probe (TCI). Acquisition parameters for 2D SOFAST-HMQC experiments were 1,024 (^1^H) and 256 (^15^N) complex points for a sweep width (SW) of 16 p.p.m. (^1^H) and 26 p.p.m. (^15^N), with 16 scans. For 3D HNCACB and CBCACONH experiments, 1,024 (^1^H), 72 (^15^N) and 96 (^13^C) complex points for a SW of 16 p.p.m. (^1^H), 26 p.p.m. (^15^N) and 70 p.p.m. (^13^C), with 16 scans. Standard delays, pulse- and gradient shapes were used^65^,^66^. NMR spectra were zero-filled to four times the number of real points and processed by apodization with a sine-modulated window function and baseline correction in all dimensions. Backbone assignments were performed with CARA (Computer-Aided Resonance Assignment, Institute for Molecular Biology and Biophysics, Zürich, Switzerland). NMR spectra of SUV-bound protein samples were obtained with 50 μM of ^15^N isotope-enriched α-Syn at 30 °C for better signal-to-noise ratios.

In-cell NMR spectra were acquired on 600 and 900 MHz Bruker Avance spectrometers, equipped with cryogenically cooled proton optimized triple resonance >‘inverse' NMR probe (TCI). All in-cell spectra were acquired at 10 °C. Parameters for 2D SOFAST-HMQC experiments were 1,024 (^1^H) and 256 (^15^N) complex points, 128 scans and 60 ms recycling delays for α-Syn in A2780 cells, and 1,024 (^1^H) and 128 (^15^N) complex points, 2,048 (2 K) scans and 60 ms recycling delays for α-Syn in RCSN-3 cells. SW's were 16 p.p.m. (^1^H) and 26 p.p.m. (^15^N) for (U)-^15^N isotope-enriched oxidized α-Syn and 16 p.p.m. (^1^H) and 14 p.p.m. (^15^N) for ^15^N/^13^C methionine-isotope-enriched α-Syn. ^13^C decoupling was used for ^15^N/^13^C methionine-isotope-enriched α-Syn. One-dimensional (1D) ^1^H–^15^N SOFAST-HMQC spectra were acquired with identical settings and 4,096 (4 K) scans. NMR spectra were processed by zero-filling to four times the number of real points and by apodization with a sine-modulated window function and baseline correction in all dimensions.

Time-resolved NMR experiments were performed on 600 and 750 MHz Bruker Avance spectrometers, equipped with cryogenically cooled proton optimized triple resonance >‘inverse' NMR probe (TCI). 1D and 2D ^1^H–^15^N SOFAST-HMQC experiments were acquired at 25 °C. All time-resolved 2D NMR spectra were recorded with 1,024 (^1^H) and 128 (^15^N) complex points, 96 scans and recycling delays of 60 ms (acquisition time ∼20 min). NMR spectra were processed by zero-filling to four times the number of real points and by apodization with a sine-modulated window function and baseline correction in all dimensions. Processing and visualization of NMR spectra were performed with TOPSPIN 3.1 (Bruker) and SPARKY (UCSF). ^1^H–^15^N mean-weighted chemical shift differences were obtained using the equation [(Δ*δ*H)^2^+(Δ*δ*N/10)^2^]^1/2^ (ref. 67), where Δ*δ*H and Δ*δ*N are the ^1^H and ^15^N chemical shift differences between reduced and oxidized α-Syn. SSP scores were calculated using Cα and Cβ chemical shifts as input according to Marsh *et al*.^68^. For Met-oxidized α-Syn, the Cβ of Met1 and Met5 was not included in the analysis because of the direct influence of the sulfoxide moieties on Cβ chemical shift values. A five-residue window average was used in both cases. Positive SSP values ranging from 0 to 1 and negative values from 0 to −1 represent the propensities of α and β structures, respectively. NMR signal intensity ratios (*I/I*_0_) were determined for ∼110 unambiguously assigned, nonoverlapped cross-peaks of oxidized and reduced α-Syn by quantifying their intensities in the presence (*I*) and absence (*I*_0_) of SUVs. Error bars in the time-resolved reaction profiles represent the average experimental noise of NMR signals used to determine the repair kinetics.

### Oxidation repair and phosphorylation kinetics

Methionine sulfoxide repair kinetics were delineated by measuring signal intensities and/or volumes of unambiguously assigned, well-resolved cross-peaks reporting on the oxidation states of Met1, Met5, Met116 and Met127 (ref. 66). These were as follows: Met1, Asp2, Met5, Lys6, Gly7 or Leu8 for Met1 and Met5; Met116 for Met116; and Met127 and Ser129 for Met127. To fit the kinetic constants for the repair processes at Met1 and Met5, we considered a mechanism that involves the formation of three different species, that is, oxidized Met1 and Met5 (*M*1_ox_*M*5_ox_), oxidized Met1 and reduced Met5 (*M*1_ox_*M*5_red_) and reduced Met1 and Met5 (*M*1_red_*M*5_red_), as is evident from the three sets of NMR cross-peaks observed in our lysate time course experiments (Fig. 3a):


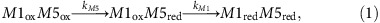


In this model, Met1 and Met5 are independently reduced according to Michaelis–Menten kinetics with *K*_M_ of Met5<<*K*_M_ of Met1. Moreover, to simplify the fitting procedure, we assumed the apparent kinetic constants *k*_*M*1_ and *k*_*M*5_ to be constant in the range of employed substrate concentrations (0–25 μM), which serves a good approximation along the published *K*_M_ values of MSRA and MSRB enzymes, that is, ∼100 μM (refs 53, 69, 70).

Thus, we solved the following differential equations:


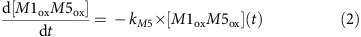



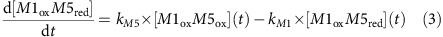



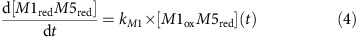


For the fitting of experimental data we used:


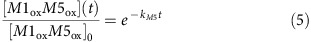



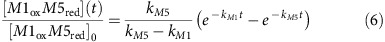



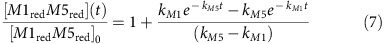


Phosphorylation rates were obtained by measuring cross-peak intensities and/or signal volumes of unambiguously assigned, well-resolved NMR cross-peaks reporting on the individual phosphorylation states of residues Tyr39, Tyr125, Tyr133, Tyr136 and Ser129 in the different time-resolved NMR spectra^66^. These were Asn122 and Asp121 for Tyr125 phosphorylation; Glu139 and Ala140 for Tyr136 phosphorylation; Tyr133 for Tyr133 phosphorylation; Leu38, Val40 or Gly41 for Tyr39 phosphorylation; and Ser129 for Ser129 phosphorylation.

### In-cell NMR samples

In-cell NMR samples were prepared following a recently developed electroporation protocol^37^. Briefly, A2780 and RCSN-3 cells were grown at 37 °C, 5% CO_2_, in T175 culture flasks in RPMI 1640 (Millipore, FG1215) and DMEM-Ham's F-12 (Biochrom, FG4815) growth media, respectively, supplemented with 10% fetal bovine serum (Biochrom, S0615) until 80% confluence. In all, 50–100 × 10^6^ cells were collected for each in-cell NMR sample. Cells were detached with mild trypsin treatment (Biochrom, L2153) and resuspended in 2 ml of electroporation buffer (100 mM sodium phosphate, 15 mM HEPES, 5 mM KCl, 15 mM MgCl_2_, 2 mM ATP and 2 mM reduced glutathione, pH 7.0). Fully oxidized, uniformly ^15^N isotope- or ^15^N methionine-enriched α-Syn (500 μM) was added to electroporation mixtures and aliquots (100 μl) were used for individual electroporations with an Amaxa nucleofector (Lonza) according to the manufacturer's instructions. Cells were re-seeded in 15-cm culture dishes and allowed to recover for 4 h at 37 °C (CO_2_ incubator). Cells were washed 3 × with pre-warmed PBS and collected with mild trypsin treatment, washed 2 × with pre-warmed growth media to remove excess trypsin and resuspended in pH-stable Leibovitz's L-15 medium (Gibco, 31415-029) supplemented with 10% D_2_O. Finally, cells were transferred to NMR tubes for in-cell NMR experiments.

### Cell lysates

Typically, 20–50 × 10^6^ cells were grown to ∼80% confluence at 37 °C, 5% CO_2_, in T175 flasks and were detached with mild trypsin treatment, washed twice with PBS and resuspended in NMR buffer with 1 × protease inhibitor mix (Complete EDTA-free, Roche). Cells were centrifuged for 5 min at 130*g* and the supernatant discarded. The cell pellet was lysed with five freeze–thaw cycles on dry ice/ethanol. Soluble lysate fractions were obtained by centrifugation at 16,000*g* for 10 min at 4 °C. Quantitative cell lysis was confirmed with Trypan Blue staining. Protein concentrations were determined using Bradford assays (Bio-Rad). Lysates were used immediately or aliquoted, snap-frozen in liquid nitrogen and stored at −80 °C until use. Repeated thawing and freezing of lysate aliquots was avoided to preserve enzyme activities^66^. Stock lysates were prepared at ∼15 mg ml^−1^ total protein concentrations and, unless otherwise specified, they were used at 10 mg ml^−1^ final concentrations. Samples for western blotting were boiled for 15 min in SDS-Laemmli buffer (Bio-Rad). Mammalian cell lines were as follows: A2780 (human, ovarian, Sigma-Aldrich), B65 (rat, neuroblastoma, Sigma-Aldrich), SH-SY5Y (human neuroblastoma, provided by Jan Bieschke, MDC-Berlin), RCSN-3 (rat *substantia nigra*, provided by Pablo Caviedes, University of Chile)^36^, SK-N-SH (human neuroblastoma, Sigma-Aldrich) and HeLa (human cervical cancer, Sigma-Aldrich).

### Repair reactions in cell lysates

Lysate concentrations were adjusted to 10 mg ml^−1^ (total protein) with NMR buffer and supplemented with 20 mM dithiothreitol (DTT) (Applichem)^31^. Repair reactions were carried out with 25 μM (U-)^15^N or ^15^N/^13^C methionine-selective isotope-enriched, N-terminally acetylated α-Syn in duplicates at 25 °C. Consecutive 2D NMR experiments were started immediately after addition of α-Syn to cell lysates. Reaction volumes were 150 μl in 3-mm diameter Shigemi tubes.

### Western blotting

Samples were separated with SDS–PAGE using 12 or 4–20% gradient gels (Bio-Rad) and transferred on polyvinylidene difluoride (PVDF) membranes. Blots were fixed with 4% paraformaldehyde for 15 min and washed thoroughly with PBS. Afterwards, blots were blocked in 5% non-fat dry milk in TBS-T for 1 h at room temperature and incubated with the following primary antibodies for 12 h at 4 °C: anti-α-Syn (1:1,000 Abcam ab51252), anti-MSRA (1:1,000, Abcam ab16803), anti-MSRB1 (1:200, Abcam ab66061), anti-MSRB2 (1:500, Abcam ab101513), anti-MSRB3 (1:500, Abcam ab88731), anti-TRX (1:1,000, Abcam ab86255), anti-TRXR (1:500, Abcam ab16847) or anti-β-Actin (1:5,000, Abcam ab6276). After washing in PBS and TBS (2 × 5 min each), membranes were incubated with horseradish peroxidase-coupled secondary antibodies for 1 h at room temperature and probed using SuperSignal West Pico or Femto chemiluminescent substrate (Thermo Scientific). Luminescence was detected and quantified on a Bio-Rad Molecular Imager and with the ImageLab software. Primary data and original western blot membranes are shown in Supplementary Figs 8 and 9.

### Immunofluorescence microscopy

Immunofluorescence images of fixed cells electroporated with reduced or oxidized α-Syn were acquired on a Zeiss LSM ultraviolet confocal microscope. After electroporation, cells were recovered for 5 h at 37 °C, 5% CO_2_ on poly-L-lysine-coated 25-mm cover slips. Slides were washed with PBS and fixed in 4% paraformaldehyde for 15 min and permeabilized with 0.1% Triton-X/PBS for 3 min. After 3 × 10 min PBS washes, samples were blocked with 0.13% gelatin from cold-water fish skin (Sigma-Aldrich) for 1 h. Slides were incubated for 2 h in 0.13% gelatin with anti-α-Syn antibody (1:500 Abcam ab51252), washed 3 × 10 min with PBS and incubated with anti-Rabbit IgG Atto647 (40839 Sigma-Aldrich, 1:1,000 dilution) for 1.5 h in 0.13% gelatin. Cells were washed 3 × 10 min with PBS and stained with 2 μg ml^−1^ 4,6-diamidino-2-phenylindole (Invitrogen) in PBS for 15 min. Images were acquired at × 60 magnification.

### *In vitro* phosphorylation reactions

Fyn tyrosine kinase was purchased from SignalChem and Plk3 serine kinase from Millipore. *In vitro* phosphorylation reactions were carried out in duplicates at 25 °C with 250 U (1 U=1 pmol min^−1^) of the respective kinases and 25 μM of α-Syn in NMR buffer, at pH 7.0, supplemented with 1 mM ATP (Fermentas), MgCl_2_ and DTT according to the suppliers' instructions. Final reaction volumes were 150 μl in all cases.

## Additional information

**How to cite this article:** Binolfi, A. *et al*. Intracellular repair of oxidation-damaged α-synuclein fails to target C-terminal modification sites. *Nat. Commun.* 7:10251 doi: 10.1038/ncomms10251 (2016).

## Supplementary Material

Supplementary Information Supplementary Figures 1-9 and Supplementary Table 1.
