# Supplementary material for: Intracellular repair of oxidation-damaged α-synuclein fails to target C-terminal modification sites
Source: Nat Commun. 2016 Jan 25;7:10251. doi: 10.1038/ncomms10251 (PMC4737712; doi:10.1038/ncomms10251)
Supplement: Supplementary Information — Supplementary Figures 1-9 and Supplementary Table 1. [file ncomms10251-s1.pdf]

Supplementary Figure 1

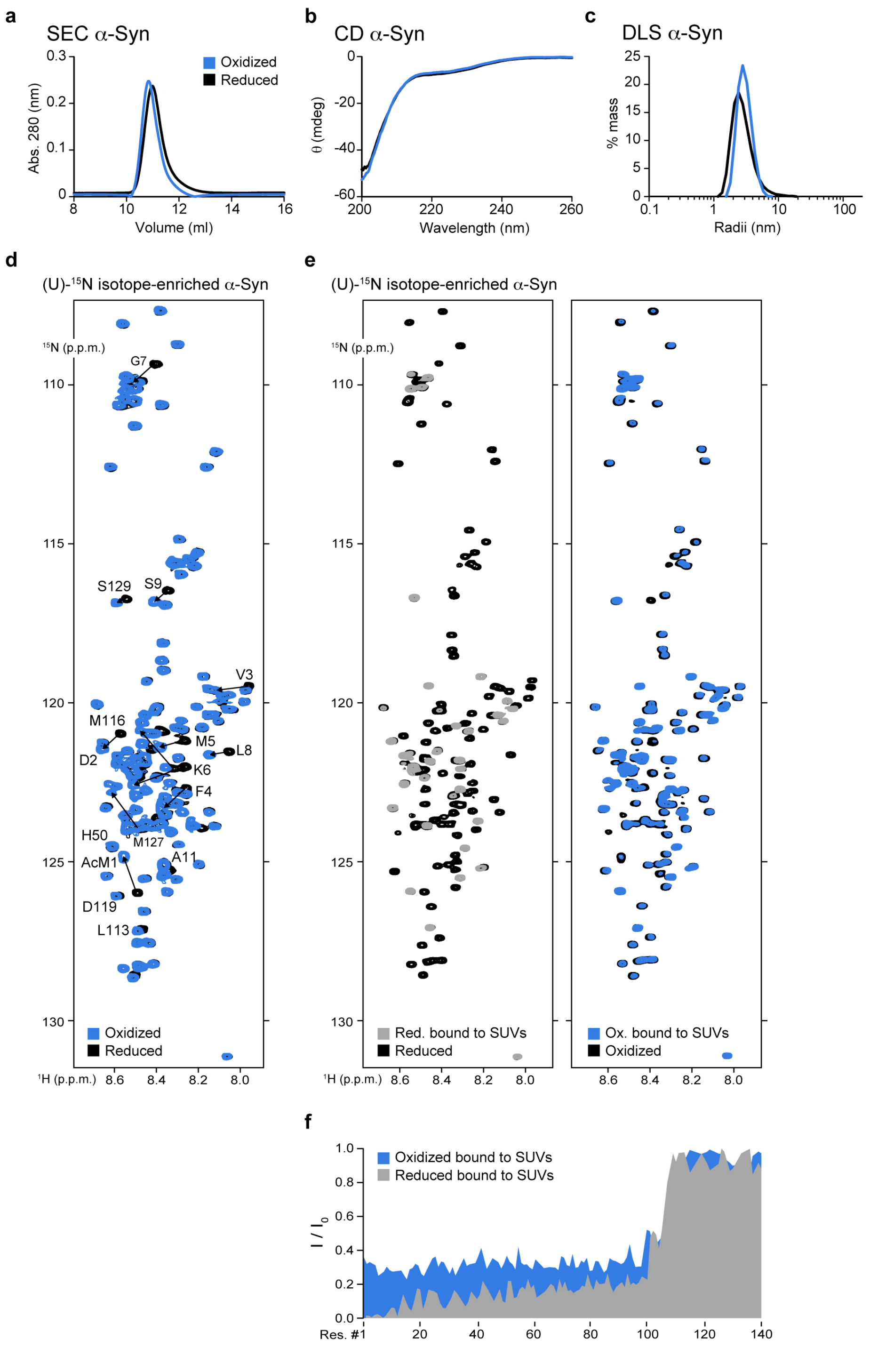

Supplementary Figure 1: Biophysical characterization of reduced and methionine-oxidized, N-terminally acetylated α-Syn (a) Size exclusion chromatography (SEC) profiles, **(b)** circular dichroism (CD) spectra, **(c)** dynamic light scattering (DLS) traces and **(d)** overlay of 2D NMR spectra of reduced (black) and methionine-oxidized (blue) recombinant N-terminally acetylated α-Syn. **(e)** Overlay of 2D NMR spectra of reduced (left) and oxidized (right) α-Syn in the absence (black) and presence of a 360-fold excess of small unilamellar vesicles (SUVs) reconstituted from pig brain polar lipid extracts. Grey and blue

contours depict reduced and oxidized α-Syn, respectively, plotted at identical contour levels. **(f)** Residue-resolved signal intensity ratios (I/I_0) upon addition of SUVs to reduced (grey) and oxidized (blue) α-Syn (360:1). Regions of α-Syn that interact with SUVs i.e. residues 1-100, are identified by characteristic reductions in I/I_0 values. Note that oxidized α-Syn binds SUVs less strongly. Experiments were performed at 5 °C **(a)**, 15 °C **(b, c** and **d)** and 30 °C **(e** and **f)**.

Supplementary Figure 2

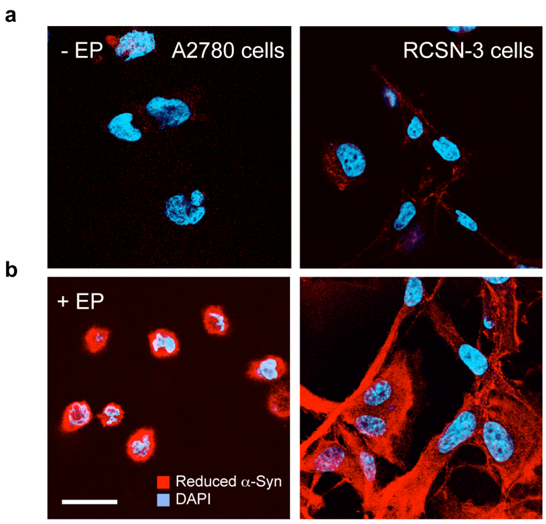

Supplementary Figure 2: Reduced α-Syn in A2780 and RCSN-3 cells (a) Immunofluorescence microscopy of A2780 and RCSN-3 cells after electroporation with buffer (-EP) and **(b)** reduced α-Syn (+EP). Images were acquired 5 h after electroporation and recovery. Size bar is 50 μm.

Supplementary Figure 3

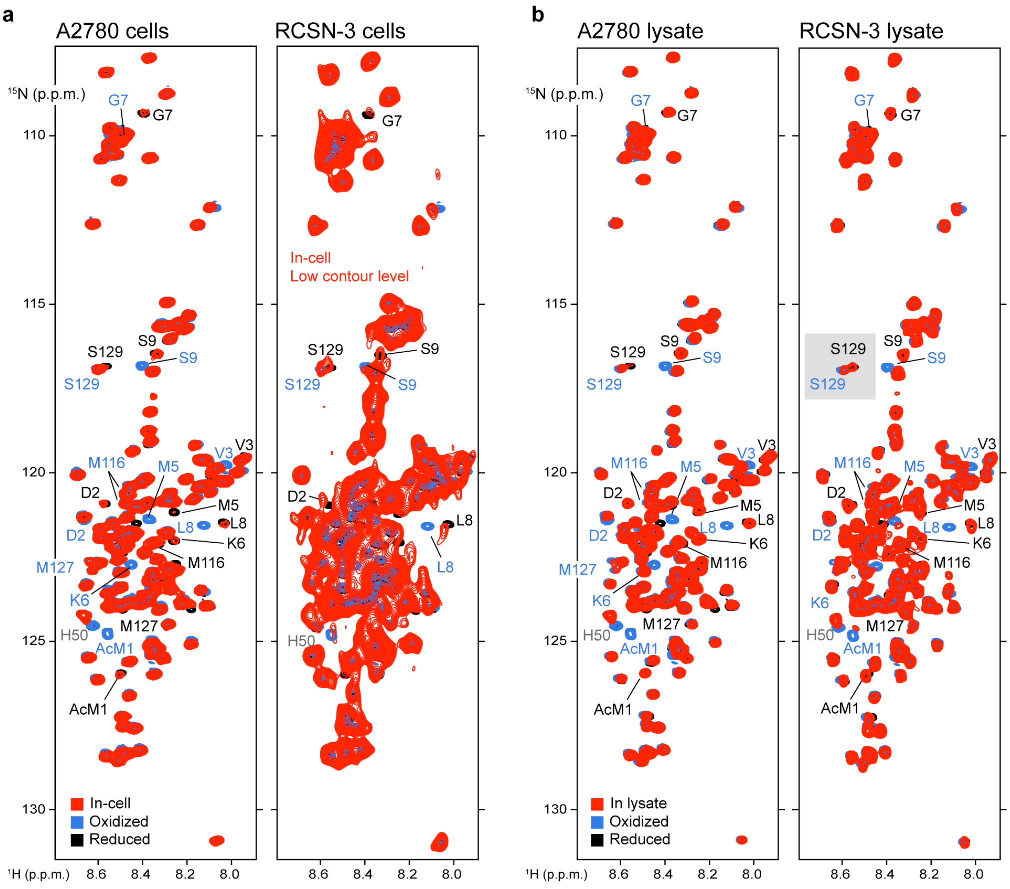

Supplementary Figure 3: NMR spectra of methionine-oxidized α-Syn in A2780 and RCSN-3 cells and lysates (a) Overlay of 2D NMR spectra of (U)-^{15}N isotope-enriched, oxidized α-Syn in A2780 and RCSN-3 cells and **(b)** upon lysis and *in situ* NMR measurements of the resulting cell slurries. Reference NMR spectra of reduced and oxidized α-Syn are shown in black and blue, respectively. Labels indicate characteristic differences in cross-peak positions between reduced and oxidized protein. The grey box in the RCSN-3 lysate spectrum highlights the split Ser129 signals indicative of the partial repair of Met127. In-cell NMR spectra were recorded at 10 °C, 5 h after electroporation and recovery.

Supplementary Figure 4

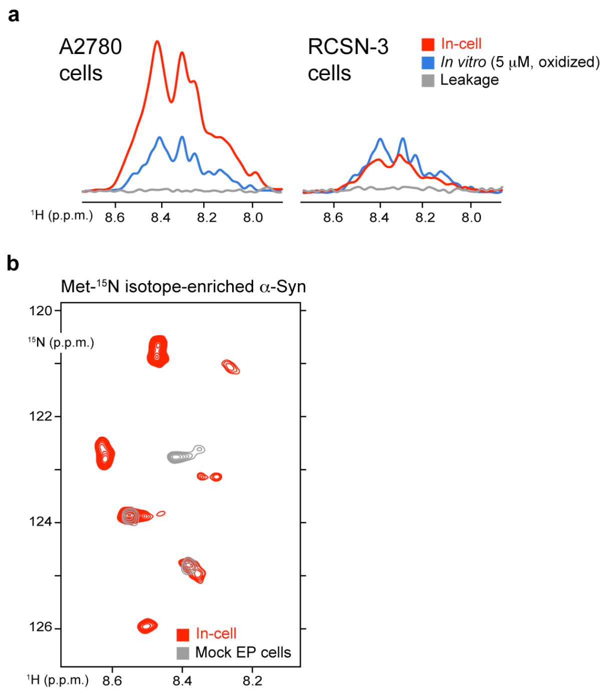

Supplementary Figure 4: Leakage and background controls of in-cell NMR samples (a) ^{15}N-edited 1D SOFAST-HMQC spectra of (U)-^{15}N isotope-enriched, oxidized α-Syn in A2780 and RCSN-3 cells (red). The blue NMR trace corresponds to a 5 μM reference sample of oxidized, N-terminally acetylated α-Syn. The grey trace is the sample supernatant collected after in-cell NMR experiments, ruling out protein leakage. **(b)** Overlay of 2D NMR spectra of A2780 cells electroporated with Met-^{15}N isotope-enriched α-Syn (red), or with buffer alone (mock, grey). Only natural abundance background signals are seen in the latter. In-cell NMR spectra were recorded at 10 °C.

Supplementary Figure 5

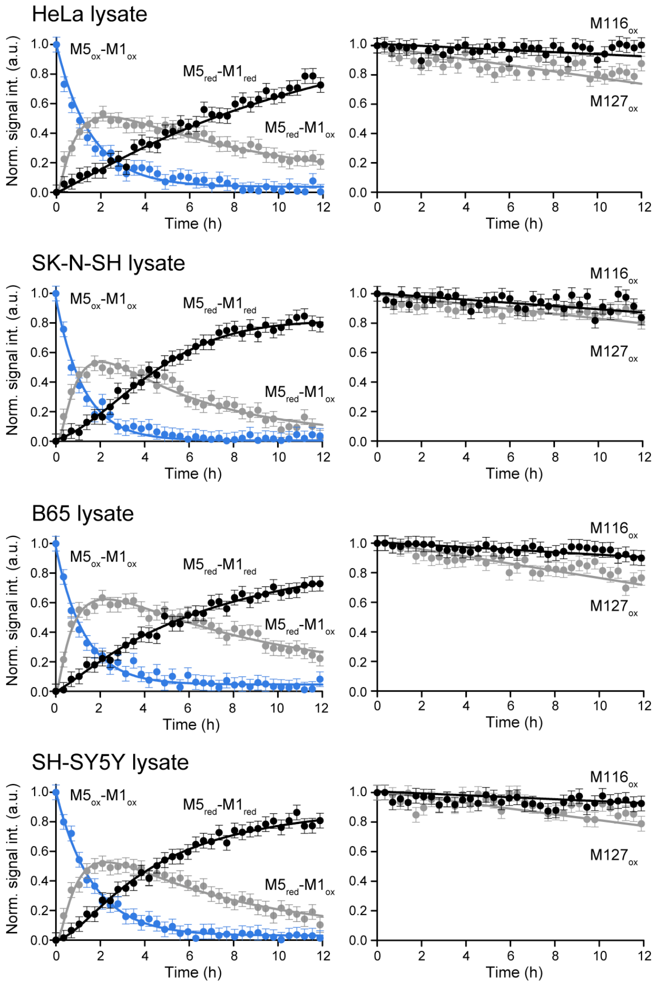

Supplementary Figure 5: Methionine sulfoxide repair kinetics in different cell lysates Real-time NMR profiles of site-selective sulfoxide repair kinetics in HeLa, SK-N-SH, B65 and SH-SY5Y cell lysates. NMR spectra were recorded at 25 °C with 25 µM of (U)-^{15}N isotope-enriched methionine-oxidized, N-terminally acetylated α-Syn in 150 µL of cell lysates (10 mg mL^{-1} total protein concentration, 20 mM DTT). Error bars represent the average experimental noise of the respective 2D NMR spectra. Lysate repair reaction properties were confirmed with two independent samples for each cell line.

Supplementary Figure 6

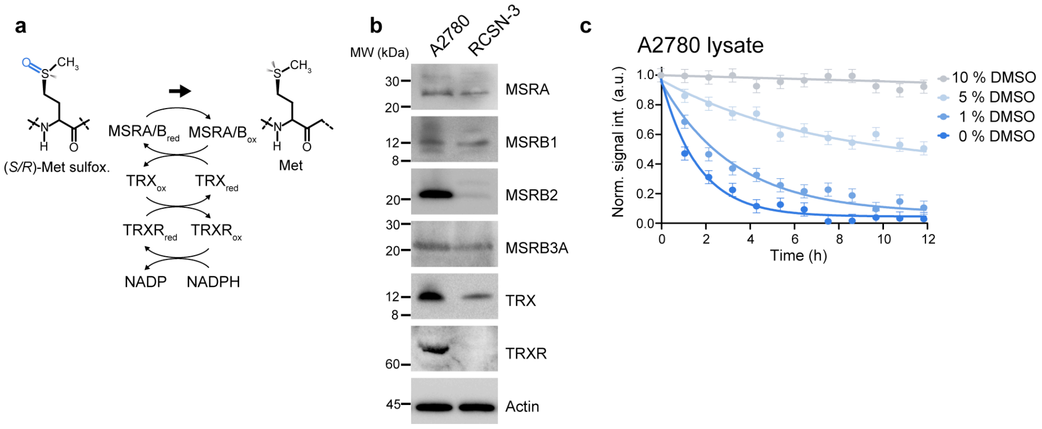

Supplementary Figure 6: Sulfoxide repair enzymes and MSR inhibition in A2780 cell lysates (a) Overview of enzyme systems required for the repair of methionine sulfoxides in cells. **(b)** Western blot analysis of endogenous MSR, thioredoxin (TRX) and thioredoxin reductase (TRXR) enzymes in human A2780 and rat RCSN-3 cells. Absence of MSRB2 and TRXR signals in RCSN-3 cells are due to the human isoform specificities of the respective primary antibodies. **(c)** Real-time NMR profiles of changes in Met5 sulfoxide repair kinetics in A2780 cell lysate with increasing amounts of the MSR inhibitor DMSO. Lysate NMR spectra were recorded at 25 °C with 25 μM of (U)-^{15}N isotope-enriched Met oxidized α-Syn in 150 μL of lysates (10 mg mL^{-1} total protein concentration, 20 mM DTT). Error bars represent the average experimental noise of respective 2D NMR spectra. Lysate repair reaction properties were confirmed with two independent samples for each DMSO concentration.

Supplementary Figure 7

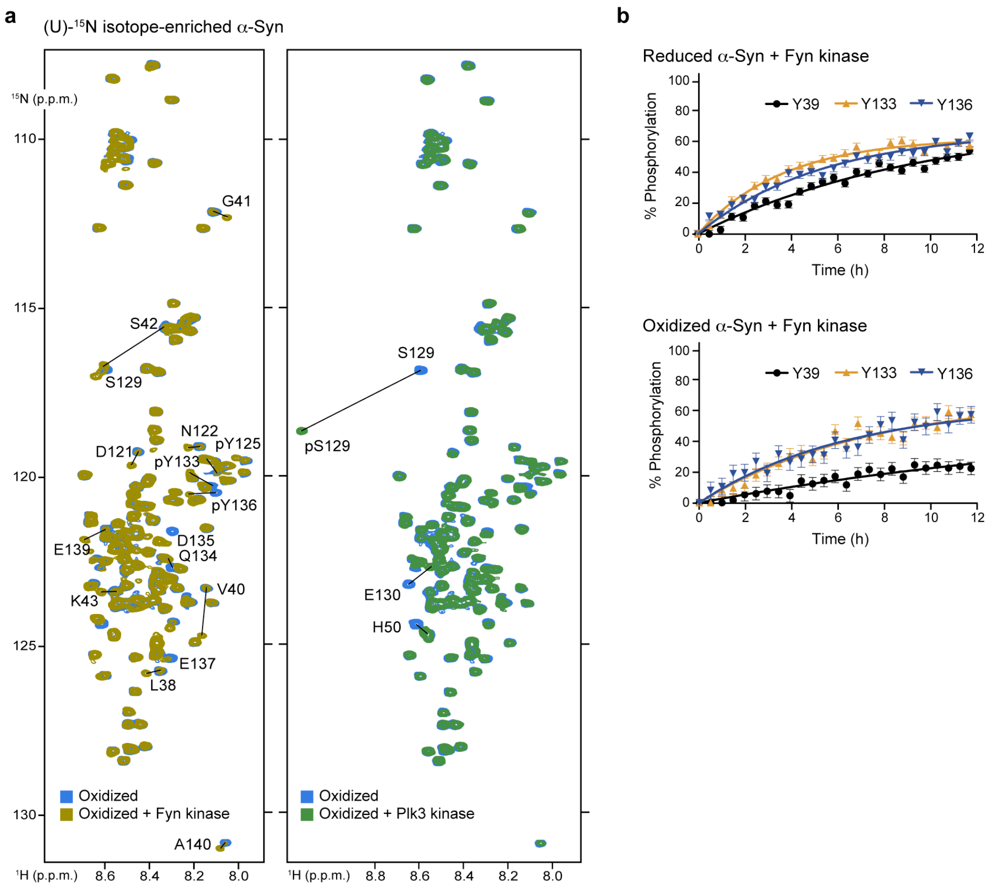

Supplementary Figure 7: NMR characteristics of α-Syn phosphorylation with Fyn and Plk3 (a) Overlay of 2D NMR spectra of methionine-oxidized, N-terminally acetylated α-Syn (blue) modified with recombinant Fyn- (brown) and Plk3-kinase (green). Phosphorylation-induced chemical shift changes of modified and neighboring protein residues are annotated. **(b)** Real-time NMR profiles of Fyn-mediated Tyr39, Try133 and Tyr136 phosphorylation of reduced (top panel) and methionine-oxidized, N-terminally acetylated α-Syn (bottom panel). Reactions were carried out with 250 U of recombinant Fyn, at 25 °C. Phosphorylation of Tyr125 is shown in **Figure 4b**. Error bars represent the average experimental noise of the respective 2D NMR spectra. Fyn phosphorylation behaviors were confirmed with two independent samples.

Supplementary Figure 8

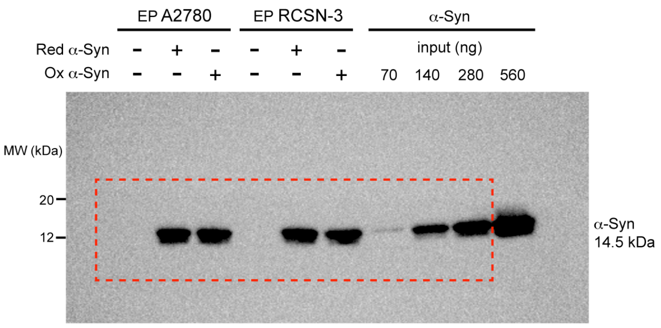

Control lanes: recombinant α-Syn, human, aa 1-140, 14.5 kDa.
Primary antibody: anti-α-Syn (Abcam 51252), monoclonal, tissue culture supernatant, dilution 1:1000

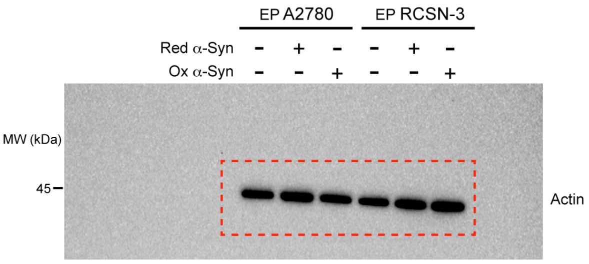

Primary antibody: anti-β-Actin (Abcam 6276), monoclonal, from ascites, dilution 1:5000

Supplementary Figure 8. Original Western blot membranes used for Figure 2b. Western blots of lysates of A2780 and RCSN-3 cells electroporated with reduced or oxidized α-Syn (top). N-terminally acetylated, reduced α-Syn was used as the input control. The bottom panel shows the upper part of the same blot probed for Actin. Dotted red lines indicate the portions of the blots shown in **Figure 2b**.

Supplementary Figure 9

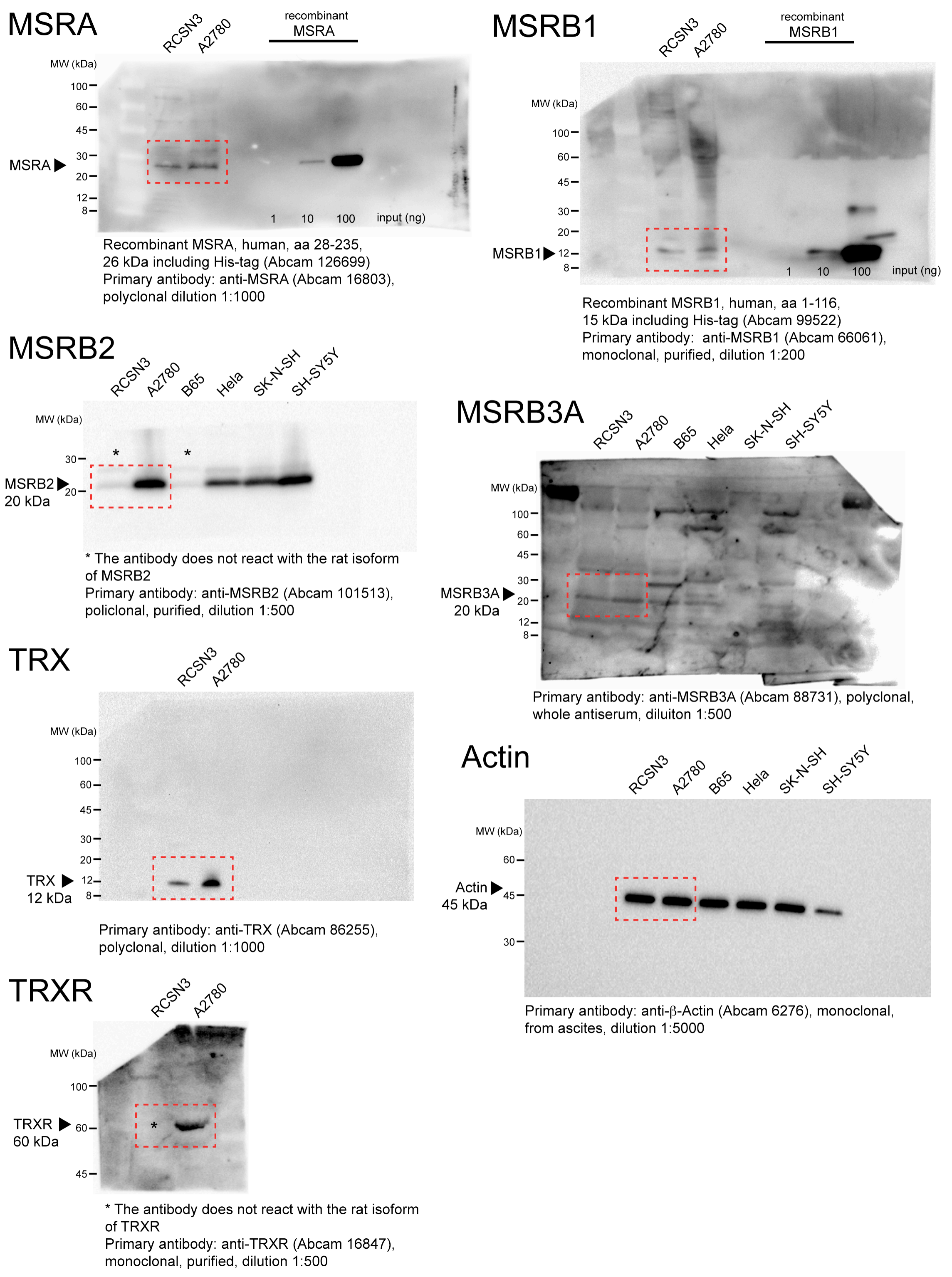

Supplementary Figure 9. Original Western blots membranes used for Supplementary Fig. 6b. Western blots of endogenous MSRs, thioredoxin (TRX), thioredoxin reductase (TRXred) and actin proteins. For MSRA, MSRB1, TRX and TRXR only A2780 and RCSN-3 lysates were used. MSRB2, MSRB3A and Actin were made with lysates from RCSN-3,

A2780, B65, Hela, SK-N-SH and SH-SY5Y cells. Dotted red lines denote the portions of the blots shown in **Supplementary Fig. 6b**.

Supplementary Table 1

**Apparent kinetic rate constants for Met1 and Met5 sulfoxide repair in mammalian cell lysates.**			
**Cell line**[a]	**Apparent kinetic rate constants**[b]		
	k_{M1}	k_{M5}	k_{M5}/k_{M1}
A2780	0.37 ± 0.02	1.03 ± 0.10	2.51 ± 1.34
RCSN-3	0.33 ± 0.05	0.83 ± 0.09	2.51 ± 1.34
HeLa	0.20 ± 0.04	0.58 ± 0.04	2.90 ± 1.63
SK-N-SH	0.25 ± 0.03	0.71 ± 0.10	2.84 ± 1.50
B65	0.18 ± 0.01	0.71 ± 0.03	3.94 ± 1.23
SH-SY5Y	0.23 ± 0.02	0.56 ± 0.01	2.43 ± 0.51

[a]In all cases, total protein concentrations of cell lysates were adjusted to 10 mg mL^{-1} and supplemented with 20 mM DTT. 25 μM of (U)-^{15}N labeled α-Syn was added for time-resolved NMR measurements. Final volume: 150 μL. All reactions were performed at 25 °C.

[b]Apparent kinetic rate constants k_{M1} and k_{M5} are expressed in h^{-1}.
